# Exploring the Shift in International Trends in Mobile Health Research From 2000 to 2020: Bibliometric Analysis

**DOI:** 10.2196/31097

**Published:** 2021-09-08

**Authors:** Jianfei Cao, Yeongjoo Lim, Shintaro Sengoku, Xitong Guo, Kota Kodama

**Affiliations:** 1 Graduate School of Technology Management Ritsumeikan University Ibaraki Japan; 2 Department of Business Administration Ritsumeikan University Ibaraki Japan; 3 Department of Innovation Science Tokyo Institute of Technology Tokyo Japan; 4 Institute at School of Management Harbin Institute of Technology Harbin China

**Keywords:** mobile health, digital health, digital medicine, bibliometric analysis, journalology, data visualization, co-occurrence analysis, research trends, mental health, mHealth, paradigm, innovation, smartphone, research, trend, literature, bibliometric, review, app, cooperation, development, public health, health policy, self-management, adolescent

## Abstract

**Background:**

Smartphones have become an integral part of our lives with unprecedented popularity and a diverse selection of apps. The continuous upgrading of information technology has also enabled smartphones to display great potential in the field of health care.

**Objective:**

We aimed to determine the future research direction of mobile health (mHealth) by analyzing its research trends and latest research hotspots.

**Methods:**

This study collected mHealth-related literature published between 2000 and 2020 from the Web of Science database. Descriptive statistics of publication trends of mHealth research were determined by analyzing the annual number of publications in the literature and annual number of publications by country. We constructed visualization network maps of country (or regional) collaborations and author-provided keyword co-occurrences, as well as overlay visualization maps of the average publication year of author-provided keywords to analyze the hotspots and research trends in mHealth research.

**Results:**

In total, 12,593 mHealth-related research papers published between 2000 and 2020 were found. The results showed an exponential growth trend in the number of annual publications in mHealth literature. JMIR mHealth and uHealth, the Journal of Medical Internet Research, and JMIR Research Protocols were the 3 top journals with respect to number of publications. The United States remained the leading contributor to the literature in this area (5294/12,593, 42.0%), well ahead of other countries and regions. Other countries and regions also showed a clear trend of annual increases in the number of mHealth publications. The 4 countries with the largest number of publications—the United States, the United Kingdom, Canada, and Australia—were found to cooperate more closely. The rest of the countries and regions showed a clear geographic pattern of cooperation. The keyword co-occurrence analysis of the top 100 authors demonstrated 5 clusters, namely, development of mHealth medical technology and its application to various diseases, use of mHealth technology to improve basic public health and health policy, mHealth self-health testing and management in daily life, adolescent use of mHealth, and mHealth in mental health. The research trends revealed a gradual shift in mHealth research from health policy and improving public health care to the development and social application of mHealth technologies.

**Conclusions:**

To the best of our knowledge, the most current bibliometric analysis dates back to 2016. However, the number of mHealth research published between 2017 and 2020 exceeds the previous total. The results of this study shed light on the latest hotspots and trends in mHealth research. These findings provide a useful overview of the development of the field; they may also serve as a valuable reference and provide guidance for researchers in the digital health field.

## Introduction

In recent years, smartphones have become popular in many countries; especially in high-income countries such as the United Kingdom and the United States—as of September 2019, the smartphone penetration rate is as high as 80% [1]. With the popularity of smartphones, the richness of smartphone app functions and the anytime-anywhere operability provide more opportunities for health promotion, especially in the medical field [2,3]. Services for medical and public health supported by mobile devices is defined as mobile health (mHealth). The outbreak of coronavirus disease 2019 (COVID-19) in 2020 has exposed the lack of medical resources in many countries [4-7]. In this context, mHealth apps can monitor body information, such as heart rate, as well as behavioral information, such as real-time acceleration, through smartphones, smartwatches, and other mobile devices. This can enable people to check their health status at any time and provide medical staff with more reference data [8-10]. Therefore, the development of mHealth can alleviate the shortage of medical resources to a certain extent [11,12]. The great potential shown by mHealth in the medical field has received attention from researchers in many countries [13]. A focus for an increasing number of researchers is to determine how further developments in the mHealth field can reasonably create more social value; therefore, it is necessary to have an in-depth understanding of current research trends and hot spots in mHealth.

Bibliometrics can quantify comprehensive textual information to provide numerical statistics on the development process of a particular topic [14]. The quantified numerical information can also help scholars identify the future trends of a subject [15]. Bibliometrics is widely used in academics, specifically for the in-depth analysis of journal papers [16,17]. Recently, researchers have developed many tools that meet the needs of bibliographic analysis and enrich the bibliographic treatment, such as for the analysis of co-authors’ countries (or regions) and research institutions to elucidate the collaboration between different regions or research institutions [18-20], the extraction of keywords for co-occurrence analysis to identify research hotspots [21,22], and keyword clustering to identify the main research directions in a field [23,24]. Thus, bibliometrics plays an important role, both in providing an overview of the past and to provide predictive information.

Currently, there are only a few papers on bibliometric analyses of mHealth literature. Sweileh et al [13] searched Scopus for mHealth papers between 2006 and 2016 and found that most keywords were related to diabetes, medication adherence, and obesity. This study also found an exponential growth in mHealth literature.

Shen et al [25] collected 2704 papers related to mHealth from the Web of Science database as of 2016. Although different from the database searched by Sweileh et al [13], the results of the 2 studies were similar in that both found the United States to be the most active country in mHealth research worldwide; they also showed an exponential growth trend in publications on mHealth in the Web of Science. By identifying the keywords, Shen et al [25] classified the research hotspots in mHealth research into the following 4 main areas: (1) patient engagement and patient intervention, (2) health monitoring and self-care, (3) mobile device and mobile computing, and (4) security and privacy.

Another bibliometric analysis [26] of mHealth literature, published in 2020, focused on papers related to mHealth apps. A total of 2802 papers published between 2000 and 2019 were collected from the Web of Science. The current state of research, research trends, hotspots, and coauthorship networks showed that the United States, England, Australia, and Canada were the most productive countries for mHealth apps research and the hot topics of mHealth apps research formed 5 clusters: (1) technology and system development of mobile health apps, (2) mobile health apps used in mental health, (3) mobile health apps used as mobile health tools in telemedicine, chronic disease, and medication adherence management, (4) mobile health apps used in health behavior and health promotion, and (5) mobile health apps used in disease prevention via the internet.

However, a gap—between 2017 and 2020—in bibliometric analysis of mHealth research remains. Both Sweileh et al [13] and Shen et al [25] found that there was an exponential growth trend up until 2016; therefore, it can be expected that the number would have grown substantially from 2017 to 2020. In fact, the number of publications in the mid-2017 to 2020 period surpasses the previous total. Therefore, a renewed bibliometric analysis of mHealth research from 2000 to 2020 was necessary. The period 2000 to 2020, instead of only 2017 to 2020, was chosen to facilitate the calculation of logical growth curves for publications and the visualization of trends in research hotspots.

## Methods

### Data Collection

We collected metadata (paper title, abstract, author keywords, author information, country, and references [27]) on papers related to mHealth published between 2000 and 2020 from the Web of Science database. The Web of Science database was chosen because it covers a wide range of fields of study and includes 21,000 peer reviewed and high-quality journals. In addition, the Web of Science includes 6 high-impact citation databases, the Science Citation Index extension, Social Science Citation Index, and many regional databases [28] in its core collection. Thus, the Web of Science database was considered to be appropriate for the bibliometric analysis.

**Figure 1:**
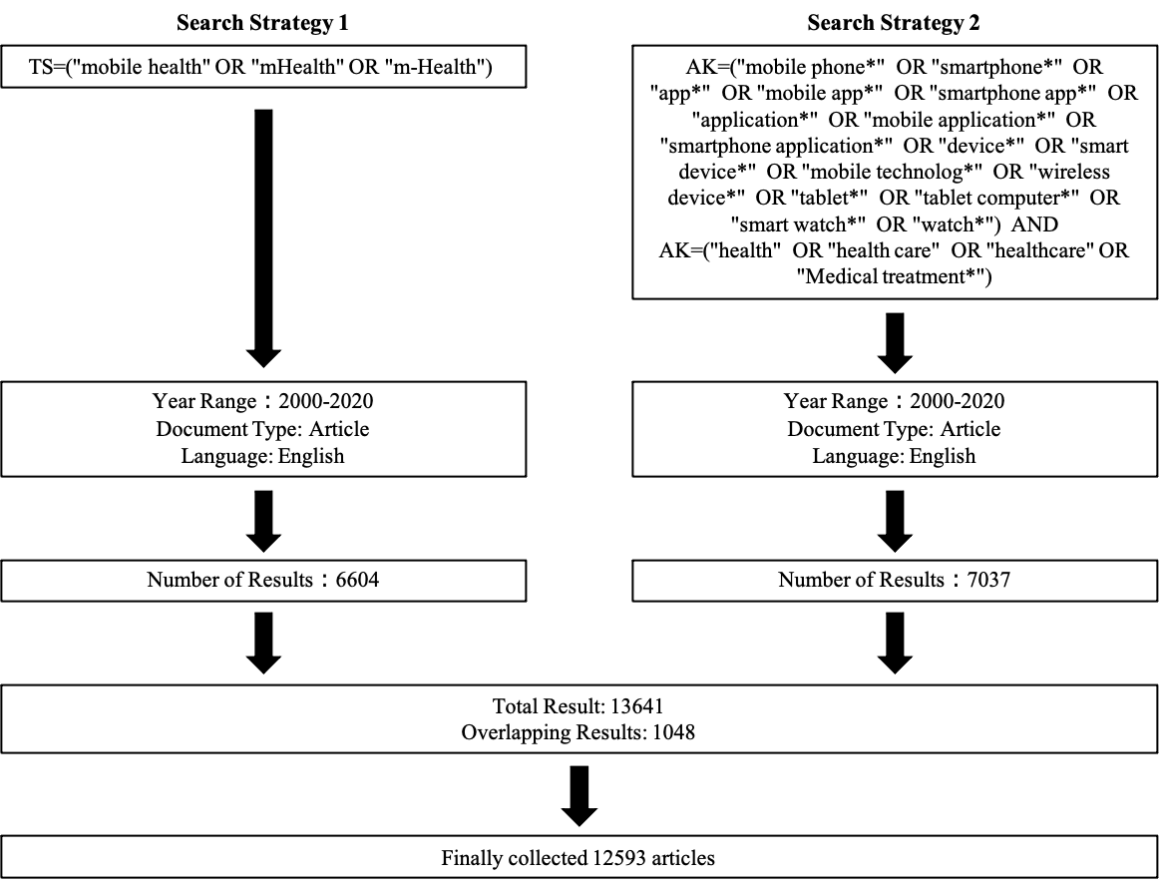
Data collection strategy for mHealth research bibliometric analysis. AK: author-provided keywords; TS: topic search.

We conducted searches using mHealth and its synonyms as search-topic keywords (in titles, abstracts, and author-provided keywords) to find potential publications related to mHealth; however, this simple approach has a major limitation. As Sweileh et al [13] suggested, many researchers might not identify their papers as focusing on mHealth though the papers are mHealth-related. Therefore, a second search strategy was also used. Given that mHealth depends on mobile devices, we searched for author-provided keywords related to both mobile devices and mHealth (smartphone, mobile phone, etc) and general health (health, health care, etc). Author-provided keyword searches were used instead of topic searches because the latter may have led to the inclusion of papers that did not emphasize the study of mobile devices and health, whereas the former represents keywords chosen by authors to highlight the contents of their papers. Thus, we determined that searching for author-provided keywords would be more appropriate to collect articles related to mHealth. Both search strategies were conducted for the period from 2000 to 2020, and only papers published in English were retrieved (Figure 1). We implemented the search on March 2, 2021. The results from both strategies were aggregated, and duplicates were removed.

### Data Analysis

We used VOSviewer (version 1.6.15) for data analysis. In bibliometric analysis, mapping and clustering techniques can provide insight into network structure and are usually used together [28,29]; however, these 2 techniques were developed independently and rely upon different ideas and assumptions. Waltman et al [30] proposed a unified mapping approach and clustering, which is used in VOSviewer [31]. This tool has been used in bibliometric analyses in many fields [32,33].

The annual number of publications, the annual growth rate, *AGR*; relative growth rate, *RGR*; doubling time, *DT*; and the growth curve of publications were calculated to observe publication trends in mHealth literature using Excel (version 2013; Microsoft Inc). In the growth curve, *x* is the number of years of growth since 2000, and *y* is the cumulative number of publications. We examined the coefficient of determination (*R*^2^) to confirm the explanatory power of the growth curve. *AGR* was defined as the percentage change in the number of publications per year and is calculated with the following formula: AGR = [(N_2_ – N_1_) / N_1_] * 100, where *N* is the annual number of publications. *RGR* was defined as the growth rate of the cumulative number of publications per unit of time and was calculated with the following formula [13,34]: RGR = [(lnTN_2_ – lnTN_1_) / (T_2_ – T_1_)] * 100, where *T* is the year and *TN* is the cumulative number of publications. *DT* was defined as the number of times the number of publications double in 1 year and was calculated with the following formula [13,34]: *DT = 0.693/RGR*.

In addition, we analyzed the publication trends by country (or region) and the distribution of publications by journal. Using VOSviewer, we created bibliometric maps for social networks, based on countries and regions, to identify international partnerships in the mHealth field.

In this study, we did a co-occurrence analysis using author-provided keywords in VOSviewer to elucidate research hotspots in the mHealth field. We set the minimum number of co-occurrences to 50. The keywords *mHealth* and *smartphone* (as well as keywords with a similar meaning) appeared more frequently because of the search strategy and took up a large weight in the co-occurrence network graph. Such keywords were considered to influence the distribution of the remaining keywords; hence, we removed the keywords used in the search strategy that appeared in the results, to focus the results on valuable research-topic buzzwords. We then extracted the top 100 keywords and mapped them into a keyword co-occurrence network. The top 100 author-provided keywords were superimposed and visualized according to the average publication year to determine the changes in research hotspots of mHealth over time. The node size indicates the number of times the author’s keyword appeared, and the color of the node changes gradually, according to the average publication year.

## Results

### mHealth Research Publications

**Figure 2:**
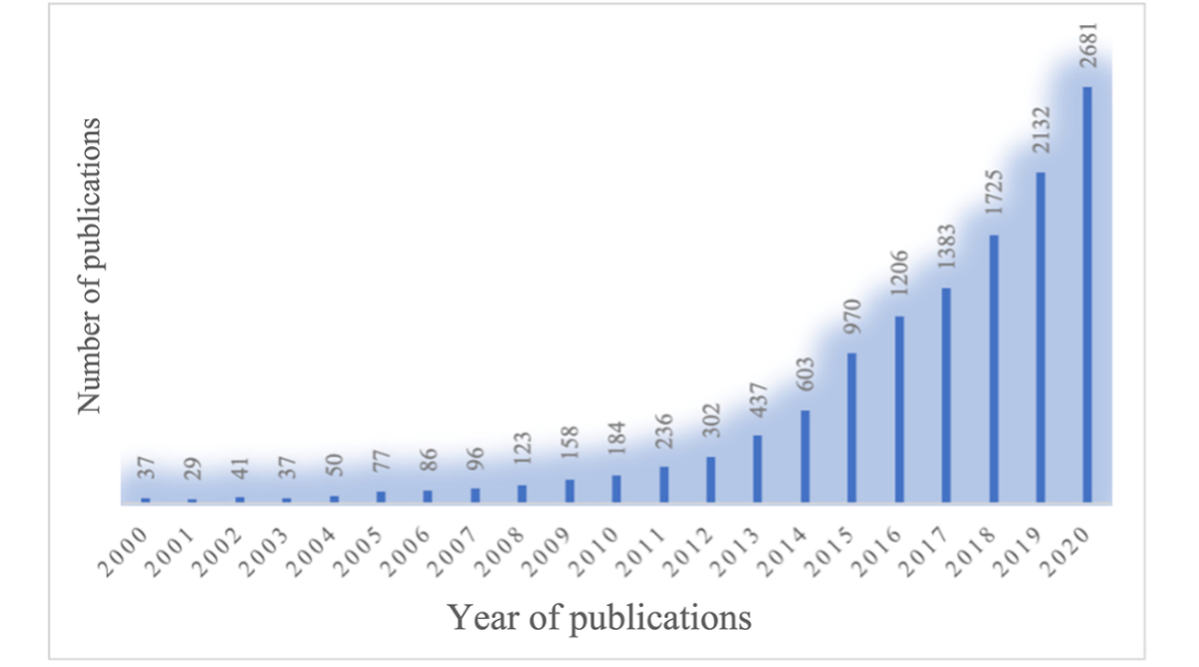
Number of publications in mHealth literature between 2000 and 2020.

**Figure 3:**
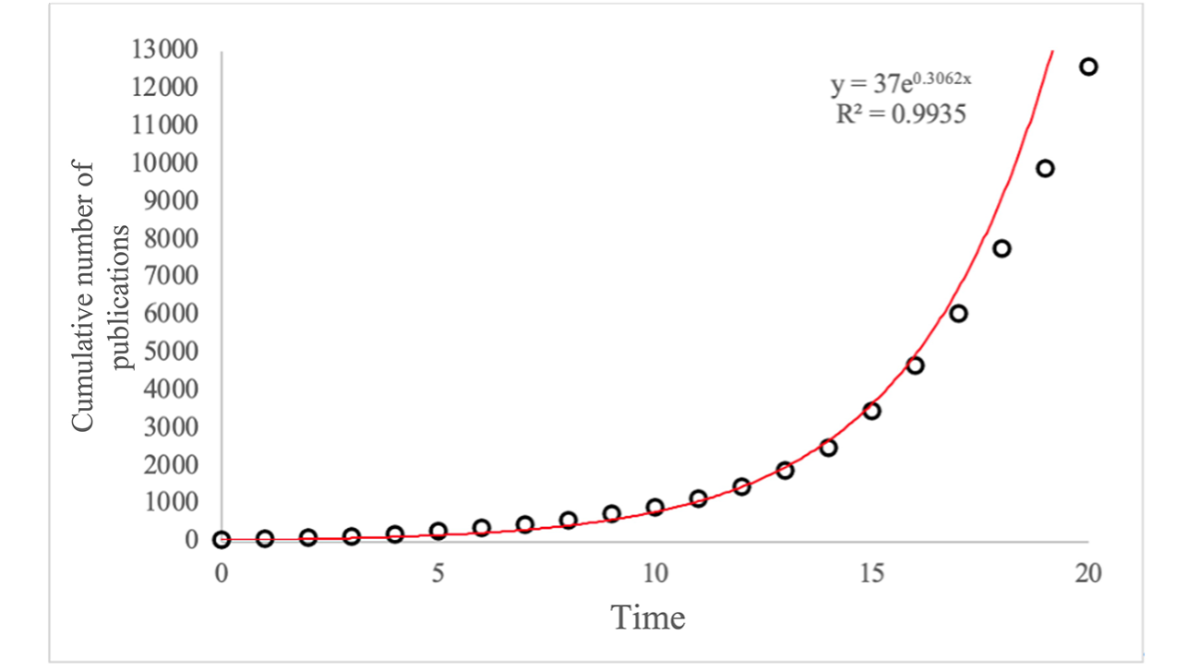
Growth curve of the cumulative number of publications in mHealth literature.

Through the first search strategy, 6604 search results were obtained, and through the second strategy, 7037 search results were obtained. After removing 1048 documents; there were 12,593 remaining (Figure 1). The number of publications related to mHealth has been increasing since 2004 (Table 1, Figure 2, and Figure 3) and has demonstrated an approximately exponential growth trend. By fitting an exponential function equation, the growth curve can be represented by *y*=37e^0.3062^*^x^*, with *R*^2^=0.9935. Specifically, the year 2015 was a flashpoint. The number of documents published in 2015 increased by 366 compared to 2014, and the annual growth rate reached 61%, becoming the highest annual growth rate in 20 years. *RGR* dropped from 58% in 2001 to 30% in 2003 and then stabilized at 28% (SD 5%). *DT* increased from 1.2 in 2001 to 2.3 in 2003 and then stabilized at 2.6 (SD 0.5). The stability of *RGR* and *DT* demonstrates the exponential growth trend [13,34] of the number of publications and confirms that the curve in Figure 3 is exponential, which indicates that the field of mHealth is increasingly receiving attention from scholars.

**Table 1:** Descriptive statistics of the collected mHealth literature.

Year	Publications, n	Annual growth, n	*AGR*^a^ (%)	*RGR*^b^ (%)	*DT* ^c^	Cumulative total, n
2000	37	N/A^d^	N/A	N/A	N/A	37
2001	29	–8	–22	58	1.2	66
2002	41	12	41	48	1.4	107
2003	37	–4	–10	30	2.3	144
2004	50	13	35	30	2.3	194
2005	77	27	54	33	2.1	271
2006	86	9	12	28	2.5	357
2007	96	10	12	24	2.9	453
2008	123	27	28	24	2.9	576
2009	158	35	28	24	2.9	734
2010	184	26	16	22	3.1	918
2011	236	52	28	23	3.0	1154
2012	302	66	28	23	3.0	1456
2013	437	135	45	26	2.6	1893
2014	603	166	38	28	2.5	2496
2015	970	367	61	33	2.1	3466
2016	1206	236	24	30	2.3	4672
2017	1383	177	15	26	2.7	6055
2018	1725	342	25	25	2.8	7780
2019	2132	407	24	24	2.9	9912
2020	2681	549	26	24	2.9	12,593

^a^*AGR*: annual growth rate.

^b^*RGR*: relative growth rate.

^c^*DT*: doubling time.

^d^N/A: not applicable.

### Publishing Trends and Cooperation Among Countries and Regions

**Figure 4:**
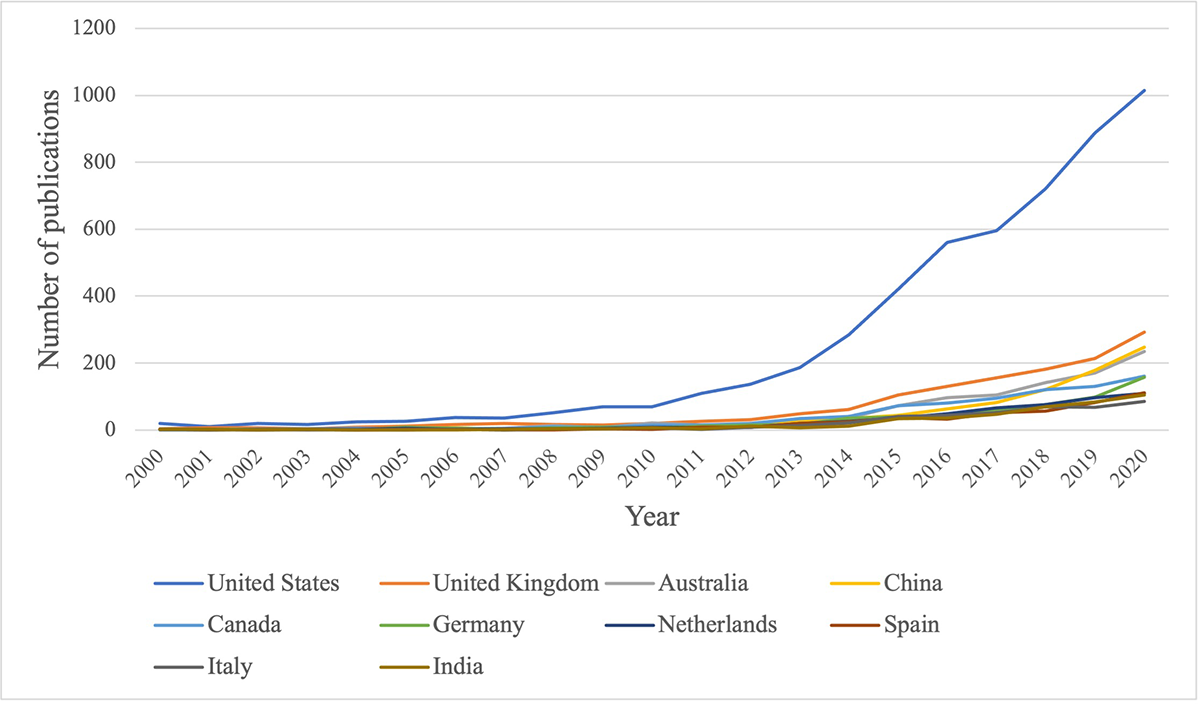
Comparison of the growth trends of mHealth-related research publications in various countries between 2000 and 2020. Due to research cooperation between scholars of different nationalities, some papers have been counted more than once.

We found that scholars from 166 countries and regions contributed to publications on mHealth (Multimedia Appendix 1). The United States had the largest number of publications (5294/12,593, 42.0%), followed by the United Kingdom (1372/12,593, 10.9%), and then Australia (979/12,593, 7.8%), China (842/12,593, 6.7%), and Canada (828/12,593, 6.6%) (Table 2). Compared with that of other countries, the growth curve of the United States shows explosive growth (Figure 4); mHealth received more attention, early on, from scholars in the United States which continued throughout the period. All countries and regions show growth, though not as high as that of the United States.

**Table 2:** Top 10 contributing countries in mHealth literature between 2000 and 2020.

Rank	Country and territory	Publications (n=12,593), n (%^a^)
1	United States	5294 (42.0)
2	United Kingdom	1372 (10.9)
3	Australia	979 (7.8)
4	China	842 (6.7)
5	Canada	828 (6.6)
6	Germany	583 (4.6)
7	The Netherlands	526 (4.2)
8	Spain	445 (3.5)
9	Italy	426 (3.4)
10	India	424 (3)

^a^Due to research cooperation between scholars of different nationalities, some papers have been counted more than once.

**Figure 5:**
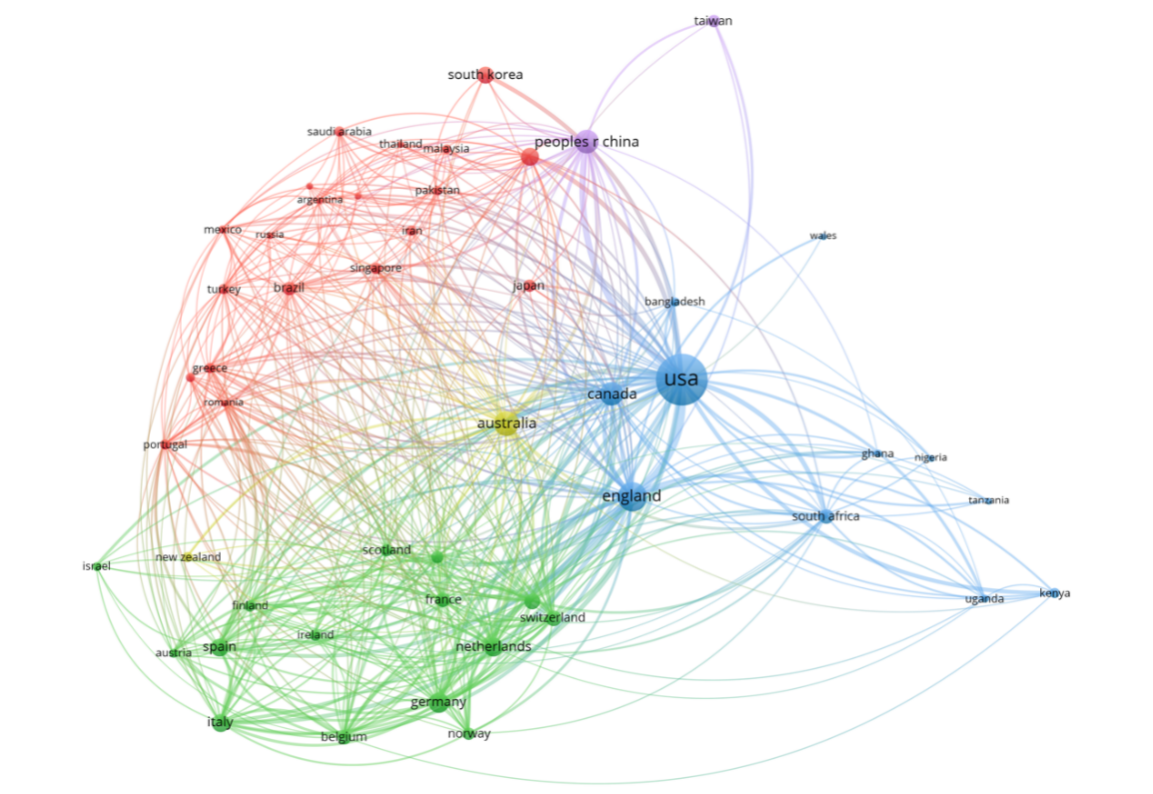
Visual network diagram of cooperation between countries or regions. The size of the circles indicates the number of publications. The larger the circle, the greater the number of publications. The length and thickness of the links between the circles indicate the strength of partnerships between countries. Asian countries and regions represented by the red cluster and the European countries and regions represented by the green cluster.

Usually, the closer the two circles, the thicker the links and the stronger the relationship (between the countries). Different colors indicate different clusters, and circles belonging to the same cluster usually have similar properties or characteristics [31]. All countries had a cooperative relationship with the United States (Figure 5). Of the top 5 countries, in terms of the number of publications, United States, the United Kingdom, Canada, and Australia occupy the center of the network diagram with similar distances between the nodes; these 4 productive countries have strong collaborative relationships. Furthermore, it is evident from the location of the countries’ nodes that the cooperation between countries and regions have geographic characteristics.

### Journal Distribution

Literature related to mHealth was distributed among 3268 journals (Table 3). The Canadian *Journal of Medical Internet Research* and its sister journals *JMIR mHealth and uHealth*, *JMIR Research Protocols*, and *JMIR Mental Health* were in the top 10 journals, with respect to number of publications, and together represented 14% of all publications (1763/12,593). In addition, all of the top 10 journals, except *JMIR Research Protocols*, have an impact factor above 2.

**Table 3:** Top 10 journals, in terms of the number of mHealth publications, between 2000 and 2020.

Rank	Journal	Country	2-year impact factor (in 2019)	Publications (n=12,593), n (%)
1	JMIR mHealth and uHealth	Canada	4.31	956 (7.6)
2	Journal of Medical Internet Research	Canada	5.03	463 (3.7)
3	JMIR Research Protocols	Canada	—^a^	235 (1.9)
4	Telemedicine and Health	The United States	2.841	202 (1.6)
5	International Journal of Environmental Research and Public Health	Switzerland	2.849	145 (1.2)
6	BMC Public Health	The United Kingdom	2.69	139 (1.1)
7	JMIR Mental Health	Canada	3.54	109 (0.87)
8	International Journal of Medical Informatics	Ireland	3.025	106 (0.84)
9	BMC Medical Informatics and Decision Making	The United Kingdom	2.317	101 (0.80)
10	Sensors	Switzerland	3.275	99 (0.79)

^a^Not available.

### Author Keywords Co-occurrence Analysis

**Figure 6:**
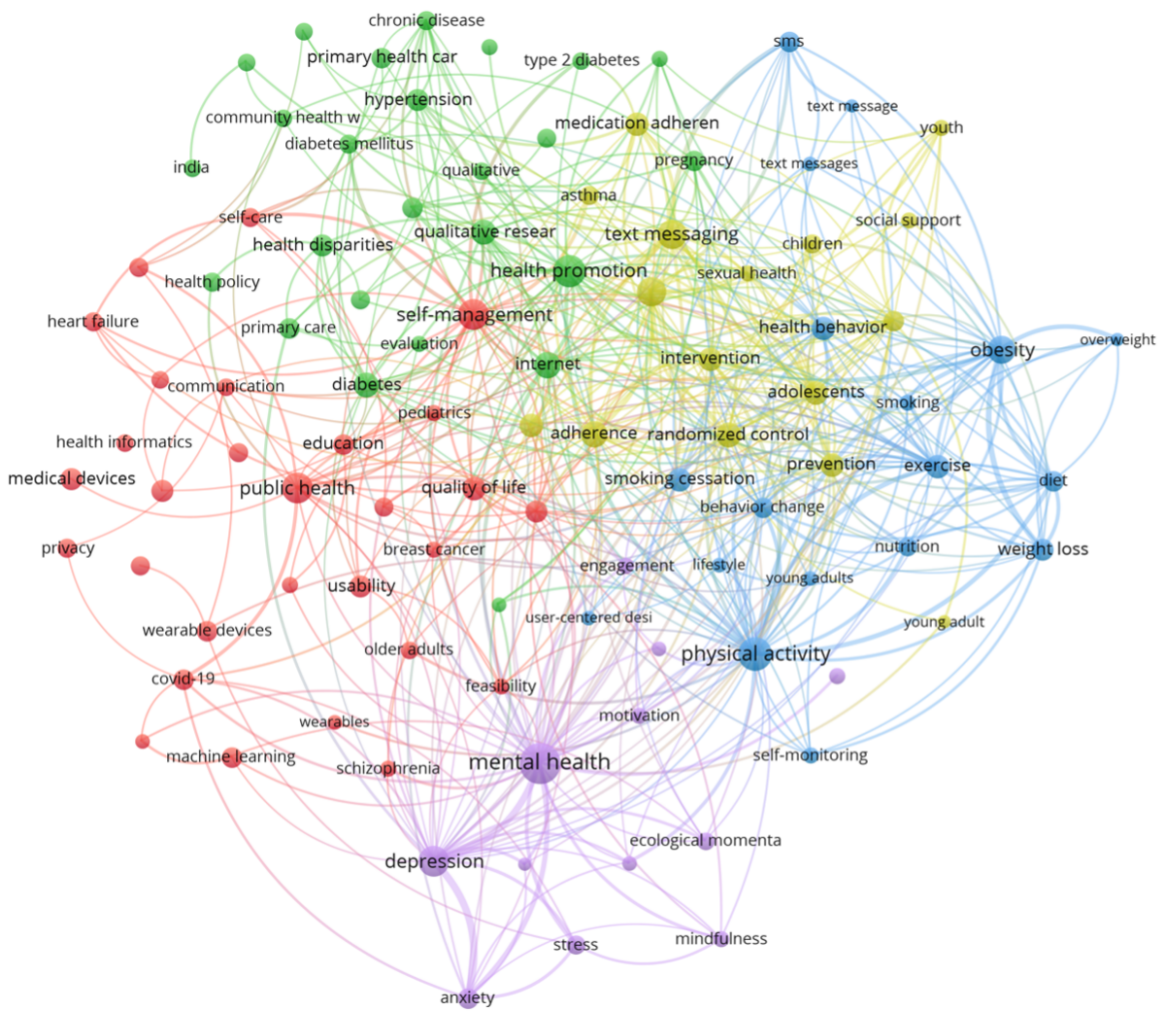
Co-occurrence network diagram of the top 100 author keywords in mHealth research between 2000 and 2020.

The top 100 keywords (Multimedia Appendix 2) were classified into 5 clusters using keyword clustering analysis (Figure 6), and the top 10 keywords by co-occurrence frequency are shown (Table 4). The average year of publication for the keywords shown in Table 4 ranged from 2015.26 to 2017.90, and the average number of citations ranged from 10.75 to 17.98. The most frequently occurring keyword was *mental health*, with a co-occurrence frequency of 449, followed by *physical activity*, with a co-occurrence frequency of 285.

**Table 4:** Top 10 author-provided keywords of mHealth research between 2000 and 2020.

Rank	Keyword	Cluster	Occurrences, n	Average year of publication	Average number of citations
1	mental health	Purple	449	2017.30	12.48
2	physical activity	Blue	285	2017.46	14.00
3	health promotion	Green	243	2015.26	14.97
4	self-management	Red	234	2017.90	10.75
5	public health	Red	232	2016.29	13.41
6	depression	Purple	227	2017.51	17.98
7	HIV	Yellow	208	2017.57	11.37
8	text messaging	Yellow	207	2016.90	13.22
9	obesity	Blue	173	2016.65	13.81
10	adherence	Yellow	157	2017.48	13.85

**Figure 7:**
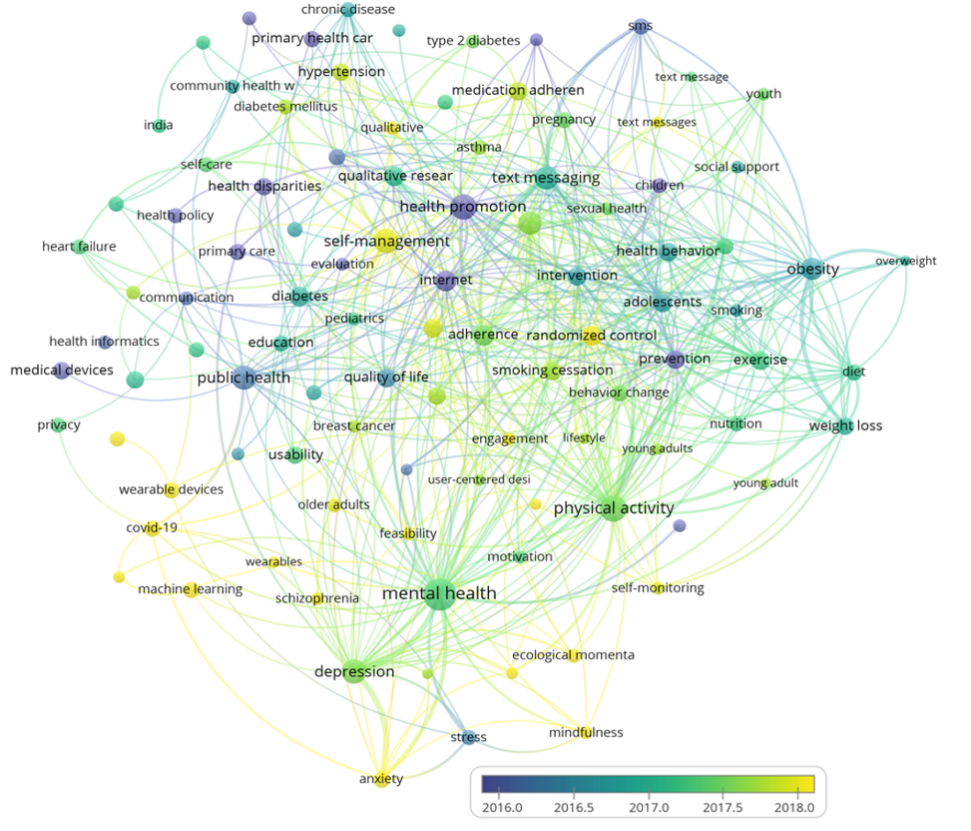
Overlay visualization maps of the average publication year of the top 100 author keywords. The more the node displays a yellow gradient, the later the average publication year of the keyword.

The average publication year range of the top 15 author-provided keywords was 2017.98 to 2020.05 (Table 5), and the occurrence range was 41 to 135 (Figure 7). Among the top 15 keywords, 8 belonged to cluster *red*, 5 belonged to cluster *purple*, 1 belonged to cluster *yellow*, and 1 belonged to cluster *green*. The average publication year range of the bottom 15 author-provided keywords was 2015.26 to 2016.19, and the occurrence range was 46 to 243. Among the bottom 15 keywords, 10 belonged to cluster *green*, 2 belonged to cluster *red*, 2 belonged to cluster *yellow*, and 1 belonged to cluster *purple*.

**Table 5:** Comparison of the top 15 and bottom 15 author-provided keywords.

Cluster			Occurrences, n		Average publication year		Keyword
**Top 15**							
	Red	86		2020.05		covid-19	
	Red	41		2019.05		artificial intelligence	
	Red	43		2018.79		wearables	
	Red	85		2018.55		machine learning	
	Purple	42		2018.54		gamification	
	Red	47		2018.48		feasibility	
	Red	87		2018.31		wearable devices	
	Purple	67		2018.26		ecological momentary assessment	
	Yellow	135		2018.24		randomized controlled trial	
	Red	69		2018.22		internet of things	
	Purple	54		2018.16		mindfulness	
	Purple	45		2018.09		sleep	
	Purple	93		2018.05		anxiety	
	Green	59		2018.04		qualitative	
	Red	55		2017.98		schizophrenia	
**Bottom 15**							
	Green	243		2015.26		health promotion	
	Green	95		2015.35		primary health care	
	Green	81		2015.40		health policy	
	Green	55		2015.45		evaluation	
	Yellow	80		2015.53		children	
	Red	103		2015.58		medical devices	
	Yellow	117		2015.79		prevention	
	Green	97		2015.82		health disparities	
	Green	152		2015.84		internet	
	Green	50		2015.94		focus groups	
	Green	87		2015.97		primary care	
	Green	67		2016.00		developing countries	
	Purple	46		2016.07		well-being	
	Red	59		2016.15		health informatics	
	Green	84		2016.19		health education	

## Discussion

### Principal Results

#### Publishing Trends of mHealth Literature

The emergence of mHealth is a great innovation in the rapid development of information technology. It has circumvented the obstacles of location and medical resources of traditional health care, making health care more accessible to a wider range of people. The growth trend for mHealth literature published between 2000 and 2020 was exponential, which suggests that, when mHealth first started, acceptance was low, the number of users was small, and research on mHealth progressed relatively slowly. As the number of users of mHealth gradually increased, more and more researchers focused on this area, and the number of mHealth publications showed an increasing trend. Based on the theory of diffusion of innovation [35], the growth curve (Figure 3) coincides with the early part of the diffusion model of innovations; we can surmise that the development of mHealth technology is currently in the early stages of rapid growth.

#### International Trends

A comparison with the bibliometric analysis [25] of mHealth research up to 2016 shows that the United States remains the most productive country in this field. The number of annual publications in the United States continues to show a steady growth trend. This is followed by the United Kingdom, China, Australia, and Canada, which are also experiencing rapid growth in their publication trends.

The 4 most productive countries—the United States, the United Kingdom, Canada, and Australia—had close cooperative relationships with each other. In contrast, the rest of the countries and regions showed a clear geographic pattern. Cluster *red* contains mainly of Asian countries such as Japan, South Korea, Russia, Malaysia, Thailand, and Singapore (Figure 5), and cluster *green* is composed mainly of European countries such as France, Netherlands, Germany, Spain, and Italy. It is not difficult to speculate that the specificity of the EU has led to closer research cooperation among EU countries. Cluster blue comprises African countries such as Kenya, South Africa, Ghana, Nigeria, Tanzania and 3 of the most productive countries—the United States, Canada, and United Kingdom. It can be presumed that African countries establish cooperation based on geography and have a major cooperation relationship with these 3 countries. In addition, China and Taiwan may be grouped in cluster *purple* because of the same language. Australia and New Zealand belong to cluster *yellow*, due to their close geographic locations. Therefore, we conjecture that international partnerships may be influenced by geography, regional characteristics, language, international relations, political, and economic alliances.

#### Research Hotspots

Cluster *red* contains the most author-provided keywords, comprising 29 keywords such as *artificial intelligence*, *electronic health records*, *global health*, *health informatics*, *health information technology*, *machine learning*, *medical devices*, *self-care*, and *wearable devices*. The keywords *breast cancer*, *cancer*, *covid-19*, *heart failure*, and other diseases also appeared in the list. This cluster focuses on the development of mHealth technologies and their application to various diseases. Globally, health issues such as aging populations and cancer pose a serious challenge to health care providers [36-38]. Researchers are increasingly trying to address many health issues with the use of mHealth technologies. *COVID-19* also appears among the high-frequency keywords. Importantly, the COVID-19 pandemic exposed the shortage of health care resources in several countries. The demand for telemedicine, including mHealth, has also been indirectly increased by countries promoting policies to prevent their population from going outside under social isolation measures adopted to tackle COVID-19 [39]. It is also worth noting that the keyword *privacy* appears in this cluster. Patient privacy, security in data transmission, and privacy-related health policy issues remain major barriers to the development of mHealth in both high-income and low- to middle-income countries [40].

Cluster *green* focuses on the use of mHealth technologies to improve basic public health and health policies. Some of the 25 keywords under this cluster include *health promotion*, *primary health care*, *health education*, *health policy*, *health communication*, and *community health workers*. Health care is one of the largest industries in the world. According to the World Health Organization, global health expenditure in 2017 was US $7.8 trillion, or approximately 10% of the total gross domestic product [41]. Compared to traditional health services, mHealth, which relies upon mobile devices such as smartphones, provides timely health information and fast, inexpensive access to primary care. As of 2017, mobile phone apps related to mHealth exceeded 325,000 [42]. Therefore, it is necessary to formulate corresponding health policies to ensure that mHealth technology can serve society more effectively and to provide direction for future health initiatives. The author-provided keyword *developing countries* appeared in this cluster. The development of mHealth in low- and middle-income countries faces more serious challenges than those faced in high-income countries. Although smartphones have become commonplace globally, challenges exist in terms of the cost of owning and using smartphones in low- and middle-income countries. For example, resource scarcity and other issues have forced low- and middle-income countries to reduce the budget for building mHealth and related infrastructure to allocate resources to other necessities such as potable water and food. The shortage of trained medical professionals and technical skills in low- and middle-income countries has also made the development of mHealth difficult [38]. Therefore, research focused on low- and middle-income countries remains a key research priority for the future of the field.

Cluster *blue* focuses on self-health testing and management in daily life. This cluster comprises 18 keywords such as *behavior change*, *diet*, *exercise*, *health behavior*, *lifestyle*, and *self-monitoring*. An increasing number of people are using emerging mHealth apps to improve their lifestyles and manage their health; these apps have a variety of functions. For example, people can control their daily calorie intake by recording their diet [43] or detect changes in their health by recording their weight, heart rate, and breathing rate [44,45]. In fact, the emergence of such apps has played a positive role in the popularization of mHealth. For example, mobile phone apps related to physical exercise have been combined with users’ social networks. People are more willing to use the tracking function of such apps to record their physical changes and share their exercise status with others, thereby increasing their social contacts’ motivation to exercise [46,47].

Cluster *yellow* focuses on the use of mHealth among adolescents. This cluster contains 16 keywords such as *adolescent*, *adherence*, *children*, *HIV*, *intervention*, *sexual health*, *social media*, *social support*, and *youth*. Research shows that the youth are the most prone to smartphone addiction [48,49]. There has been considerable research on the negative effects of smartphone addiction on health [50-52]. Excessive smartphone use affects sleep quality, and thus, other daily activities [48,53]. Adolescents are also a priority group for HIV prevention. mHealth apps that use social media technology make it easier for health workers to spread sexual health information more effectively, and thus, reduce the risk of HIV infection among adolescents [54]. Therefore, mHealth research focusing on adolescents is essential.

Cluster *purple* focuses on the use of mHealth in the context of mental health. It contains 12 keywords, including *mental health*, *anxiety*, *mindfulness*, *stress*, and *well-being*. The keyword *mental health* has the most frequent co-occurrence. Therefore, it can be assumed that this topic is the primary focus of researchers. Various factors influence mental health, such as past experiences [55], social stress [56], and interpersonal relationships [57]. People with mental health problems often resist talking to others [58], and even those who have undergone psychotherapy and have recovered are at high risk of reoccurrence [59]. Mental illness is a severe social problem, especially in high-income countries. For example, in Japan, the suicide rate due to depression has been high, and it has been increasing among youth in recent years [60,61]. For a country with a serious aging problem, an increase in the suicide rate among young people can incur a huge cost to the national economy. Moreover, people with depression can have poor physical health compared with that of individuals in the general population [62]. Timely intelligence technology that captures body information provided by mHealth can provide psychologists with more reference data to detect physical changes in patients through ecological momentary assessment, thus providing more guidance to patients.

#### Research Trends

Based on the clusters to which these keywords belong, we can speculate that mHealth research hotspots have gradually shifted from research on mHealth policy and the improvement of public health care to the development of mHealth technology and social apps (cluster *green* to cluster *red* and cluster *purple*). Thus, we find that the development of mHealth requires appropriate health policy as a cornerstone. However, individual governments usually develop health policies, leading to national and regional limitations in the scope of policy application. In contrast, the scope of web-based mHealth services can be global. This may also make it more difficult to regulate mHealth services; therefore, it is still necessary to continue to explore how to establish regulations for cross-border telehealth in the future. Furthermore, we note that in high-income countries, especially in the health care field, government regulatory formation is critical to the growth of the mHealth market [63]. Governmental oversight measures often limit the development of mHealth technologies and services [64]. Although the United States is absolutely central to mHealth research, health care regulations in the country may be more conservative and less susceptible to change due to the huge health care infrastructure. Conversely, mHealth policy reforms are likely to be smoother in low- and middle-income countries because they are met with less opposition and fewer infrastructural barriers [65]. Therefore, effective strategies are needed to advance regulatory reforms related to mHealth.

### Limitations

To the best of our knowledge, the results obtained in this study are the most recent available for mHealth bibliometric analysis; however, this study has some limitations. First, we developed a search strategy that included as many mHealth-related studies as possible, but we still could not guarantee the inclusion of all mHealth-related studies. Second, our search strategy collected only English-language literature, which narrowed the scope. Hence, the data results are not representative of papers and conference papers published in other languages. Finally, the data used in this study were extracted only from the Web of Science and did not include other search engines such as Scopus and PubMed. Although the Web of Science has a large enough database to ensure the accuracy of the data to a certain extent, there are still many papers that are included only in the other databases, which may have impacted the study results. For example, our finding suggest that only 175 mHealth papers were in Japan (Multimedia Appendix 1); however, many mHealth papers published in Japanese are included in the CiNii database maintained by the National Institute of Informatics in Japan. The Chinese Science Citation Database in China also contains many papers published in Chinese; therefore, future studies can include more databases and languages to make the research results more accurate and rigorous.

### Conclusions

This study reveals the latest research trends and hotspots and the current state of international collaboration in mHealth research. As previously suggested, mHealth has shown great potential in recent years for use in all aspects of our lives; however, the development of mHealth faces challenges from regulatory policies, national economies, and personal privacy. Therefore, we advise researchers in this field to work on these issues to further develop the mHealth field. We also hope that the results of this study provide valuable guidance for future mHealth research.

## Appendix

Multimedia Appendix 1 List of countries and regions that have contributed to publications on mHealth.

Multimedia Appendix 2 Details of the top 100 author keywords in mHealth research between 2000 and 2020.

## Abbreviations

COVID-19: coronavirus disease 2019
HIV: human immunodeficiency virus
mHealth: mobile health
